# Recent Advanced Deep Learning Architectures for Retinal Fluid Segmentation on Optical Coherence Tomography Images

**DOI:** 10.3390/s22083055

**Published:** 2022-04-15

**Authors:** Mengchen Lin, Guidong Bao, Xiaoqian Sang, Yunfeng Wu

**Affiliations:** School of Informatics, Xiamen University, 422 Si Ming South Road, Xiamen 361005, China; 23320201154003@stu.xmu.edu.cn (M.L.); 23320201153974@stu.xmu.edu.cn (G.B.); 23320201154016@stu.xmu.edu.cn (X.S.)

**Keywords:** optical coherence tomography, machine learning, neural networks, retinal fluid segmentation, ophthalmic diseases

## Abstract

With non-invasive and high-resolution properties, optical coherence tomography (OCT) has been widely used as a retinal imaging modality for the effective diagnosis of ophthalmic diseases. The retinal fluid is often segmented by medical experts as a pivotal biomarker to assist in the clinical diagnosis of age-related macular diseases, diabetic macular edema, and retinal vein occlusion. In recent years, the advanced machine learning methods, such as deep learning paradigms, have attracted more and more attention from academia in the retinal fluid segmentation applications. The automatic retinal fluid segmentation based on deep learning can improve the semantic segmentation accuracy and efficiency of macular change analysis, which has potential clinical implications for ophthalmic pathology detection. This article summarizes several different deep learning paradigms reported in the up-to-date literature for the retinal fluid segmentation in OCT images. The deep learning architectures include the backbone of convolutional neural network (CNN), fully convolutional network (FCN), U-shape network (U-Net), and the other hybrid computational methods. The article also provides a survey on the prevailing OCT image datasets used in recent retinal segmentation investigations. The future perspectives and some potential retinal segmentation directions are discussed in the concluding context.

## 1. Introduction

The human eyes are important organs that can sense light and provide a function of binocular color vision. With the growth of human age and the influence of external factors, the eyes are susceptible to a few retinal fluid disorders. Retinal fluid mainly contains intraretinal fluid (IRF), subretinal fluid (SRF), and pigment epithelial detachment (PED). These areas are vital biomarkers related to the diseases of age-related macular degeneration (AMD) and retinal vein occlusion (RVO). The retinal fluid occupation area can be detected and segmented from the medical images, which are considered to be useful for the distinguishing the retinal pathology.

**Figure 1:**
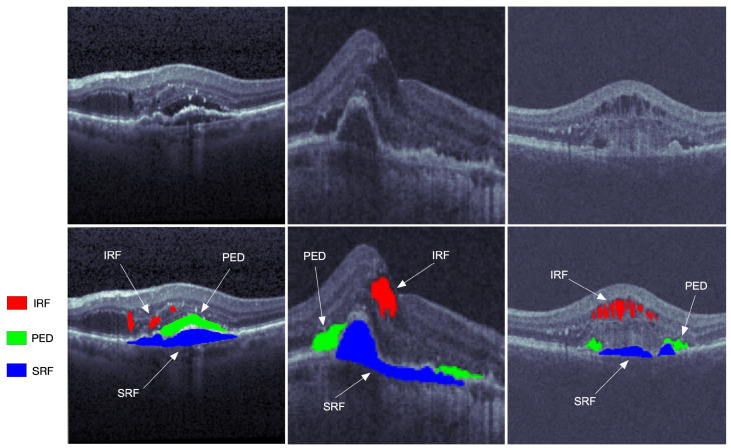
The OCT retinal images from left to right are from the three vendors of Cirrus, Spectralis, and Topcon in the RETOUCH dataset [2]. The images in the first row are not manually labeled, while the red, blue, and green segmentations on the second row represent the intraretinal fluid (IRF), subretinal fluid (SRF), and epithelial detachment (PED), respectively.

Optical coherence tomography (OCT) is one of the most widely used medical imaging technologies, which has been rapidly developing in the past three decades. Such an imaging technique was first proposed by Huang et al. [1] in 1991. The OCT technique utilizes the basic principle of the low coherent light interferometer to detect the backscattered near-infrared light and reconstruct the depth profile of the biological tissue sample. Its penetration depth is hardly limited by the transparent refractive medium of the eye. Furthermore, the OCT technique can identify the anterior segment and display the morphological structure of the posterior segment. The OCT modality is a good choice for cross-sectional imaging of the retina because its high resolution facilitates a clear visualization of retinal structures. From the images of the retinal structure obtained by the OCT modality, the fluid area can be visualized with different reflectivity measures from the surrounding tissues. The OCT image processing and analysis may help distinguish various conditions of retinal fluids and evaluate the progression of retina pathology. It has a brilliant prospect in the applications of retinal pathology diagnosis, follow-up observation, and treatment effect evaluation of intraocular diseases. Some typical segmentations of OCT images are shown in Figure 1.

Primarily located in the inner and outer nuclear layers, the IRF is regarded as separated hyporeflective cystoid pockets that could increase the overall retinal thickness. The IRF is one of the important variables of vision loss. The SRF is a hyporeflective space that corresponds to the clear or lipid-rich exudate between the neurosensory retina and the retinal pigment epithelium (RPE). The SRF is related to the AMD, and would cause the retinal detachment. As one of the main indicators of progressive disease, the PED is the separation of RPE from the Bruch’s membrane (BM), which can be subdivided into the serous, fibrovascular, and drusenoid.

With the rapid development of high-performance computer hardware and sufficient large datasets, the computer vision and deep learning methods have been dramatically improved and evolved during recent years. In comparison with the traditional neural network methods, deep learning network architectures normally contain many more hidden layers that have powerful scalability and hierarchical feature learning capability to automatically extract the morphological features from raw image data. Since 2016, the deep learning algorithms have made significant impacts on the retinal fluid segmentation based on OCT images. The most popular deep learning frameworks for the segmentation of retinal fluids include the convolutional neural network (CNN), fully convolutional network (FCN), U-shape network (U-Net), and hybrid computational methods. The major strategy of the deep learning is to identify the contours of retinal fluids, and then commonly solve a classification problem with the semantic context extracted from the OCT images.

**Figure 2:**
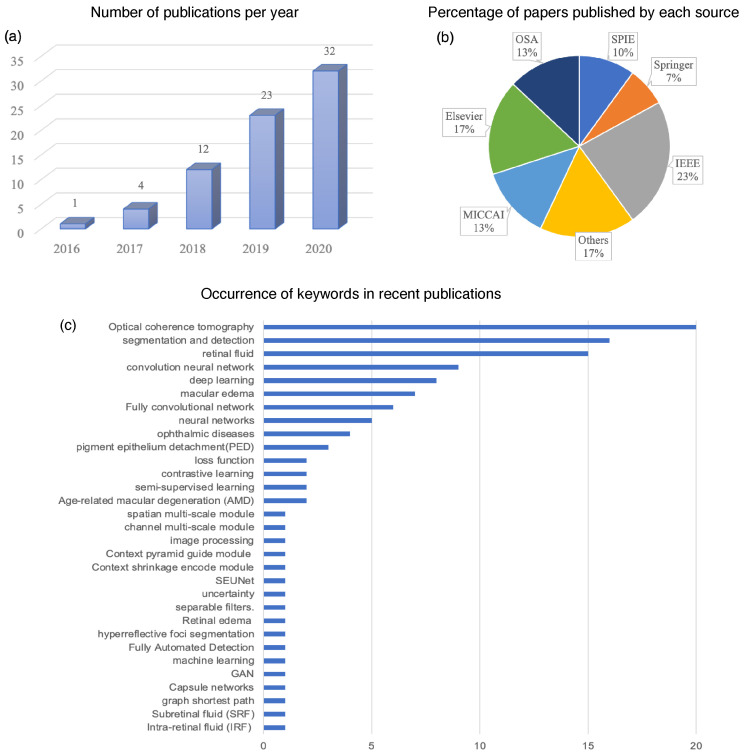
Statistics of the papers and their sources on the deep learning methods for retinal fluid segmentation published in recent years. (**a**) Number of publications on retinal fluid segmentation based on deep learning from 2016 to 2020. (**b**) Percentage of papers published by conferences and journal publishers. (**c**) Keywords listed in the recent publications.

Figure 2 shows the statistics of papers on deep learning for retinal fluid segmentation published in academia from 2017 to 2020. According to the statistics from the Web of Science database, the relevant publications on retinal fluid segmentation topics have consistently grown each year, from 1 in 2016 up to 32 in 2020. The conference paper collections are from MICCAI, ISBI, SPIE Medical Imaging, EMBC, IJO, ICDIP, and ISSPIT, and the journal sources include IEEE, Springer, OSA, and Elsevier. In general, the majority of these papers were published by IEEE and Elsevier, which accounted for about 40% of all. The keywords of these papers mainly cover OCT, deep learning, retinal fluid, segmentation, IRF, SRF, PED, AMD, and macular edema. As the retinal-fluid-segmentation-related studies were brought into primary focus, it calls for a review of the recent progress of advanced deep learning architectures and algorithms for retinal fluid segmentation. This article provides a survey on the segmentations of three retinal fluids, i.e., the IRF, SRF, and PED, in OCT images with different deep learning architectures.

## 2. Deep Learning Applications for OCT Image Segmentation

**Figure 3:**
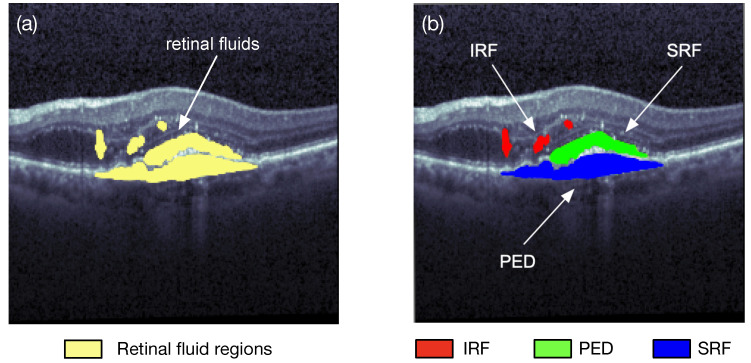
Two different types of retinal fluid segmentation on the same optical coherence tomography (OCT) image from the RETOUCH dataset [2]. (**a**) Semantic segmentation: all of the retinal fluids are segmented into yellow regions; (**b**) instance segmentation: the IRF (red), SRF (green), and PED (blue) are segmented separately.

Image segmentation is one of the research fields of computer vision. Segmentation is the process of combining the objects of interests in multiple groups in accordance with the join features in an image. Semantic segmentation and instance segmentation are the two main types of image segmentations. The semantic segmentation categorizes the objects with the same class label into a unique semantic entity, for example, grouping all humans into one class and all animals into another class. On the other hand, the instance segmentation distinguishes all instances of the same object into different classes, i.e., even some similar objects could also be marked with different labels. As illustrated in Figure 3, the semantic segmentation approach categorizes all of the retinal fluid into one class, meanwhile the instance segmentation method separates the retinal fluid as the SRF, IRF, and PED, respectively. Image segmentation can quickly locate a variety of abnormalities in medical images, such as locating pulse tumors [3] and melanoma detection [4]. It can also extract the content of interest in medical images, such as segmentations of retinal blood vessels [5] and the retinal fluids.

The most frequently used traditional imaging processing methods for retinal fluid segmentation include edge-based detection, threshold-based segmentation, and histogram-based segmentation. The edge detection method typically uses the maximum value of the first derivative of the pixels or the zero-crossing information of the second derivative to separate the boundaries of different regions. Both of the threshold-based segmentation and histogram-based segmentation utilize the grayscale features of the image to distinguish the object content from the background. The major drawback of these traditional image segmentation methods is that the different segmentation tasks would require well-devised algorithm, and sometimes the spatial information of images cannot be effectively utilized. The emerging deep learning methods have the advantages of automatically combining the low-level features of images to form more abstract high-level features, and providing higher segmentation accuracy. The following subsections provide a brief description of the popular deep learning neural network architectures for the OCT image segmentation applications.

### 2.1. Fully Convolutional Networks (FCNs)

The FCN paradigm was proposed by Shelhamer et al. [6] in 2017. The ordinary CNN uses the last of a few fully connected layers to transform the two-dimensional image matrix into one dimension to produce the class labels, or to make object localizations via regression. The major difference between FCN and CNN is their output layers. The FCN uses a fully CNN with some transposed convolutional layers to transform the height and width of intermediate feature maps back to those of the input image. The FCN produces the classification outputs in correspondence with the input image at pixel level, i.e., the channel dimension at each output pixel holds the classification results for the input pixel at the same spatial position [6]. Therefore, FCN’s symmetrical encoder–decoder structure allows the network to process input images of arbitrary sizes.

**Figure 4:**
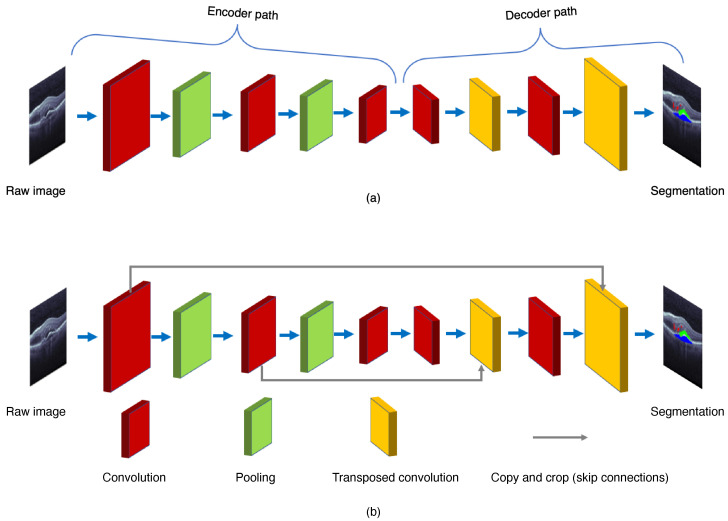
General structures of (**a**) the fully convolution network (FCN) and (**b**) the U-Net.

As shown in Figure 4, the FCN is composed of the encoder and decoder structures. The encoder is responsible for mapping an input image to the high-dimensional feature representation. On the contrary, the decoder uses the transposed convolution to upsample the feature maps, and restores them to the size of the input image when preserving the spatial information.

Either CNN or FCN has the major drawback of slow training process due to some operations, such as maxpool. Therefore, the training of either CNN or FCN usually takes a lot of time, and a high-performance graphics processing unit (GPU) is required to speed up the network computation. In addition, the FCN is an expensive architecture, because most of the cost is consumed by the fully convolutional layers in the end.

### 2.2. U-Net

To improve the segmentation performance, many other modified convolutional networks that propagate the mapped features from the encoder to the decoder have been developed. One of the most prevailing deep learning architectures for the image segmentation is the U-Net, proposed by Ronneberger et al. [7] in 2015. As shown in Figure 4, the U-Net adds the skip connections to propagate the downsampling feature map to the upsampling, and restore the semantic information through feature map splicing. The U-Net makes the image segmentation with an end-to-end setting, and has the advantages of requiring a smaller number images for training and also providing the desired localization. Recently, several effective retinal segmentation networks have utilized the U-Net or its 3D modification as their backbones. Hassan et al. [8] proposed the symptomatic exudate-associated derangement network (SEADNet), which employs two novel feature extractors based on the U-Net. Ye et al. [9] proposed the CAF-Net, which adds context shrinkage encode and context pyramid guide modules, without changing the depth of the U-Net, in order to improve the segmentation accuracy. The major drawback of the U-Net is the trade-off between localization accuracy and the context usage. Typically, larger patches of the U-Net require more max-pooling layers which may reduce the localization accuracy, whereas smaller patches lead to less context for visualization. On the other hand, the U-Net also runs slowly due to a large number of overlapping patches.

### 2.3. SegNet

With a symmetrical design of encoder and decoder, the SegNet [10] can perform the encoding and decoding with the same spatial size and same number of channels. The hierarchy decoders of the SegNet use the max-pooling indices received from the corresponding encoders to implement nonlinear upsampling of their input features. The SegNet architecture generates the sparse feature maps based on the location information, and then restores the dense feature maps through convolutions. In addition, the SegNet architecture usually adds a conditional random field (CRF) module to the ending layer in order to optimize the boundary segmentation outputs. The SegNet has the advantages of improving boundary delineation and reducing the number of end-to-end training parameters. The unsampling form the SegNet can also be effectively utilized in other encoder–decoder architectures without a significant modification.

### 2.4. DeepLab

The standard depth convolutional network would face two issues. First, the downsampling layers of a CNN may extend the sensory area; however, they would decrease the spatial resolution feature maps for the image segmentation tasks [11]. Second, the location information of the input image will decrease, and sometimes even disappear when the depth of the network changes. In order to keep both of the size and space invariance of the feature map, the DeepLab was proposed by Chen et al. [12] by incorporating the atrous convolutions, atrous spatial pyramid pooling (ASPP), and CRFs. The DeepLab has two major advantages: (1) The atrous convolution operations may speed up the GPU computing; (2) semantic segmentation accuracy can be improved with the multiscale image representations based on the ASPP.

### 2.5. Evaluation Indicators

Evaluation indicators are vital in the performance assessment of the segmentation algorithms. Appropriate indicators can be used to objectively evaluate the segmentation accuracy, classification sensitivity and specificity, versatility, and other characteristics of different medical image segmentation algorithms. Chang et al. [13] categorized the systematic evaluation methods of segmentation algorithms into three types:

- Region-based indicators;
- Distance-based indicators (e.g., Hausdorff distance, pixel distance);
- Entire image-based statistical indicators.

The Dice similarity coefficient is the most frequently used region-based indicator for segmentation accuracy evaluation [2,13], which measures the overlapping voxels between two samples, defined as

(1) $$\mathrm{Dice}=\frac{2\left|V_{p}\right|\cap \left|V_{g}\right|}{\left|V_{p}\right|+\left|V_{g}\right|}\times 100\%,$$

where ∩ is the intersection operator, and $\left|V_{p}\right|$ and $\left|V_{g}\right|$ denote the sizes of the predicted voxels and the ground truth, respectively. The Dice value typically varies in a range from 0 to 1. The Dice value equal to 1 is the ideal result that implies a perfect segmentation, whereas the Dice value equal to 0 indicates the total mismatch between the prediction and ground truth.

The Jaccard coefficient is also a region-based indicator with the definition of the ratio of the intersection of the predicted voxels and ground truth divided by their union region [13], written as

(2) $$\mathrm{Jaccard}=\frac{\left|V_{p}\cap V_{g}\right|}{\left|V_{p}\cup V_{g}\right|}\times 100\%,$$

where ∪ is the union operator. The Jaccard coefficient can measure and ensure the number of 2D pixels or 3D voxels inside the object are correctly segmented as well.

## 3. Benchmark OCT Datasets

The development of OCT image segmentation technology calls for large and context-rich datasets to train the deep learning networks. This section provides some popular public OCT datasets, which have been listed in Table 1.

### 3.1. RETOUCH Dataset

The RETOUCH dataset [2] originates from the retinal OCT fluid challenge of MICCAI 2017, in which the OCT images were marked three labels of retinal fluid, namely, IRF, SRF, and PED, respectively. Half of the patients were diagnosed with the macular edema secondary to AMD, and the other half with the edema secondary to RVO. Since the testing data in the competition are not public access, the OCT image data available for all researches actually remain as the training set. The training data consist of 70 OCT volumes in total. In particular, the numbers of volumes obtained by Cirrus (Model: 5000), Triton (Model: T-1000/T-2000), and Spectralis OCT systems are 24, 22, and 24, which have been labeled as IRF, SRF, PED, and normal, respectively. Within each volume, the numbers of B-scan images acquired with Cirrus (Carl Zeiss Meditec Inc., Jena, Germany), Triton (Topcon Corporation, Tokyo, Japan), and Spectralis (Heidelberg Engineering Inc., Heidelberg, Germany) are 128 (512 × 1024 pixels), 128 (T-2000: 512 × 885 pixels, T-1000: 512 × 650 pixels), and 49 (512 × 496 pixels), respectively. These B-scan images contain at least one liquid in a single volume. It is worth mentioning that the annotations and volume of this dataset were obtained from the Medical University of Vienna (MUV) in Austria, Erasmus University Medical Center (ERASMUS) and Radboud University Medical Center (RUNMC) in The Netherlands. The annotations were manually made on the B-scan plane by the human graders from the MUV and RUNMC clinical centers. Four graders from MUV were supervised by an ophthalmology resident and trained by two retinal specialists. Two graders from RUNMC were supervised by a retinal specialist. Most of the relevant studies covered in this review paper were carried out based on the RETOUCH dataset.

### 3.2. UMN Dataset

The UMN dataset was collected by the University of Minnesota (UMN) ophthalmology clinic. The dataset contains a total of 600 OCT B-scan images from 24 exudative AMD subjects [14]. During the acquisition process, each subject performed approximately 100 B-scan images, from which the 25 B-scan images with the largest liquid area were selected as samples to export. These scanned images were captured by Spectralis system, through an average of 12–19 frames with the resolution of 5.88 μm/pixel along the length and 3.87 μm/pixel along the width [14]. The UMN dataset includes the retinal fluid patterns of IRF, SRF, and PED. Each fluid region was manually annotated and checked by two ophthalmologists. Unfortunately, this dataset is difficult to implement segmentation algorithms, due to a large number of sub-RPE and sub-retinal fluid regions located in the eyes of exudative AMD patients. Rashno et al. [14] reported that the RPE errors in the UMN dataset would adversely affect the retinal pigment epithelium segmentation algorithm.

### 3.3. OPTIMA Dataset

The OPTIMA dataset [15] was publicly available from the cyst segmentation challenge of MICCAI 2015, and currently has been widely used for the IRF segmentation tasks. The dataset consists of 30 volumes from 4 OCT devices used in ophthalmology (i.e., Cirrus, Spectralis, Topcon, and Nidek). The dimension of each OCT volume is approximately 6 × 6 × 2 ${\mathrm{mm}}^{3}$, and the corresponding coordinate was centered on the macula. The dataset was split into two subsets of equal size: 15 volumes for training and the other 15 volumes for testing purpose. Only IRF labels for the training subset have been annotated by two different professional graders at the Christian Doppler Laboratory for Ophthalmic Image Analysis (OPTIMA), Medical University of Vienna. Either of the training or testing subset contains three volumes scanned from the Nidek (model: RS-3000 Advance) device (Nidek Inc., Tokyo, Japan), and the resting 12 volumes of each subset were equally obtained from Cirrus, Spectralis, Topcon (model: 3D OCT-2000) devices.

### 3.4. Duke Dataset

The Duke dataset [16] is a public dataset provided by Duke University. It contains 110 annotated B-scan images recorded from 10 patients with severe diabetic macular edema (DME) pathology and the annotations of eight-layer boundaries. Each patient performed 11 B-scan images, which were centered on the foveal, and 5 frames on each side of the foveal (foveal slice and scans laterally acquired at ±2, ±5, ±10, ±15, and ±20 μm from the foveal slice). The dataset also includes fluid and non-fluid regions with annotations of eight-layer boundaries. Researchers can send requests to the experts to segment the data, for the purpose of model training and algorithm testing. All of these samples were ethically licensed and special attention is paid to the anonymity of the images, which were manually labeled by two ophthalmologists for the retinal layer and fluid area.

### 3.5. HCMS Dataset

The HCMS dataset [17] is a public dataset provided by Johns Hopkins University. It includes the right-eye OCT scanning results of 35 subjects acquired with the Spectralis system. Each volume consists of 49 B-scan images (each B-scan including 1024 A-scans and each A-scan consisting of 496 pixels) and nine-layer boundary annotations of 14 healthy controls (HC) and 21 patients with multiple sclerosis (MS). Similar to the Duke dataset, the HCMS dataset only contains the manually labeled semantic fluid regions, and cannot be further subdivided. Therefore, researchers have to implement some necessary preprocessing procedures when using this dataset for validating the segmentation performance.

### 3.6. Kermany Dataset

In 2018, Kermany et al. [18] constructed the validated OCT and chest X-ray image datasets. The OCT images were scanned by the Spectralis system, and were categorized into choroidal neovascularization (CNV), DME, drusen, and normal. The Kermany dataset contains 207,130 OCT B-scan images in total. There are 108,312 OCT B-scan images recorded from 4686 patients with retinal fluid labels, including 37,206 images with CNV, 11,349 images with DME, 8617 images with drusen, and 51,140 images normal, respectively. The retinal fluid labels of the OCT images were manually annotated with a tiered grading system. The first tier of graders were undergraduate and medical students, who reviewed the diagnosis information and discarded the OCT images contaminated by severe artifacts. The second tier of graders were four ophthalmologists who independently graded the images by making records of the CNV, DME, and drusen information. The third tier of graders were two senior independent retinal specialists with over 20 years of clinical experience, who performed the final verification of image labels.

### 3.7. Other Private Datasets

In addition to the public access datasets mentioned above, some researchers use their own datasets. Schlegl et al. [19] collected 1200 OCT B-scan volumes associated with AMD (400 cases), DME (400 cases), and RVO (400 cases). Half of the OCT images were scanned by Cirrus (Model: 5000) OCT device, and the other half of images were scanned by Spectralist OCT system. The presence of intraretinal cystoid fluid and SRF were labeled by two independent experienced retinal specialists.

Gao et al. [20] used 52 B-scan volumes obtained from 35 subjects with central serous chorioretinopathy. The numbers of OCT volumes diagnosed as only PED or neurosensory retinal detachment were 6 or 23, respectively. The other 23 volumes were identified with both PED and neurosensory retinal detachment.

Lee et al. [21] carried out the automated segmentation of macular edema with the CNN on 1289 B-scan images acquired with the Spectralis OCT system. Retinal OCT image segmentations were manually performed by retinal specialists from Department of Ophthalmology, University of Washington.

Rao et al. [22] used 150 OCT volumes to test the retinal fluid segmentation based on the FCN. Each volume contained 128 B-scan images collected by the Cirrus OCT system.

The OCT dataset of Yang et al. [23] was composed of 103 OCT volumes obtained from 62 patients diagnosed as central serous chorioretinopathy. The retinal imaging was performed by the Cirrus OCT system with 128 B-scan images per volume.

In the work of Bao et al. [24], a total of 240 B-scan images from 60 AMD patients were used to test the segmentation effectiveness of the modified U-Net architectures with channel multiscale module and spatial multiscale module. The PED segmentations of each retinal OCT image were labeled under the supervision of two senior ophthalmologists.

In the study of Pawan et al. [25], they used 25 volumes (128 B-scan images per volume) of patients with central serous chorioretinopathy, which were obtained by the Cirrus (Model: 500) system. The segmentation labels for each OCT images were verified by by an expert retina surgeon with over 15 years of experience, from Pink City Eye and Retina Center, Jaipur, India.

The dataset used in the work of Hu et al. [26] contained 70, 15, and 15 volumes of OCT images for training, validation, and testing purpose, respectively. A single volume was composed of 128 B-scan images with a resolution of 512 × 1024 pixels. The SRF and PED fluids were manually annotated at the pixel level by experienced ophthalmologists.

Venhuizen et al. [27] collected 221 spectral domain OCT volumes (a total of 6158 B-scan images) from 151 AMD patients with Spectralis OCT device (model: HRA+OCT). The axial resolution of each spectral domain OCT image was 496 pixels, and the dimensions varied between 512 and 1536 pixels in the transversal direction. The number of B-scan images in each volume varied from 19 to 37, with the B-scan spacing changing from 240 to 120 μm, respectively.

## 4. Retinal Fluid Segmentation Based on Deep Learning

### 4.1. Deep Learning Paradigms Based on CNN or FCN

When computing the large dataset, deep learning algorithms can automatically extract distinct spatial features from OCT images in semantic context. Therefore, the retinal fluid segmentation results provided by the deep learning methods are usually superior to those obtained by the traditional machine learning algorithms. The deep learning architectures and paradigms for retinal fluid segmentation described in the recent publications are summarized in Table 2.

#### Modified CNN or FCN Architectures

From 2017 to 2018, several classical CNN and FCN architectures have been effectively used to segment the retinal fluid regions in OCT images. Chen et al. [28] used a faster R-CNN to segment different retinal fluids. The faster R-CNN first segmented the IRF regions. Then, the IRF segmentation results were used as the seeds to grow the 3D regions to segment the SRF, which may avoid the overfitting with the training data. Finally, the outputs from the RPE layer were utilized to segment the PED regions. Schlegl et al. [19] developed an eight-layer FCN for the end-to-end detection, and achieved excellent detection accuracy results of retinal fluids.

Venhuizen et al. [27] used two FCNs to segment the intraretinal cystoid fluids. The first FCN aimed to identify the entire retina, and the second FCN was designed to segment the retinal fluid by combining the outputs of the first FCN. During the network computation, the first FCN can be used as the additional input to the second FCN, as a constraint in the entire network training process. The network was trained by using the stochastic gradient descent with a learning rate starting at $10^{-3}$ up to $10^{-6}$, and the momentum was set to be 0.99 in order to include a large sample of the previous steps [27].

Since 2019, the deep learning architectures have become more and more complex and effective for solving the retinal fluid segmentation problems. Sanchez et al. [29] proposed a novel architecture that integrated a modified deep retinal understanding (DRIU) framework and a Pix2Pix module in the U-Net with SE-blocks (SEUNet) model. Such a deep learning architecture has the advantage of detecting small lesions with a better segmentation accuracy. Wang et al. [30] employed the DeepLab architecture to accomplish effective retinal fluid and hyperreflective foci segmentations. This model used atrous spatial pyramid pooling (ASPP) to detect macular edema and used the fully connected CRFs to refine the boundary of macular edema. Both of them reported impressive disease diagnosis results associated with the segmented retinal fluids.

Liu et al. [31] developed an EfficientNet-B5 architecture to detect DME. Such a deep learning system was trained to derive retinal thickening and IRF presence in OCT images. Then, the DME can be detected from two-dimensional color fundus photographs, based on the reference of the retinal thickness and fluid presence. Liu et al. [31] reported that the EfficientNet-B5 network can provide higher specificity and non-inferior sensitivity.

Besides the conventional CNN or FCN structures, many researchers focused on the modified deep learning networks to meet some specific retinal fluid segmentation requirements. Sappa et al. [32] designed a modified CNN architecture named RetFluidNet, which assimilated different skip-connect operations and ASPP to integrate multiscale contextual information, which achieved good segmentation performance, among all the papers counted in this paper. Girish et al. [33] preprocessed the regions of interest in OCT images to enhance the semantics, and then used a CNN with a depthwise separable convolution filter to segment the IRF regions, which can prevent the overfitting obstacle.

On the other hand, the community also paid close attention to revisions of loss functions in the deep learning networks to improve the segmentation performance. Liu and Wang [34] proposed a framework that comprised a student network and a teacher network, in order to predict the probability, contour, and distance maps simultaneously. Pawan et al. [25] made an enhancement to the existing capsule network architecture of SegCaps [35], called DRIP-Caps, which utilized the dilation, residual connections, inception blocks, and capsule pooling for the SRF segmentation, which reduces the computational complexity and the number of training parameters. They reported that the DRIP-Caps architecture might reduce the number of trainable parameters by 54.21%. Hu et al. [26] introduced the modules of ASPP and modified stochastic ASPP into the ResNet to segment the SRF and PED regions in OCT images, which can alleviate the overfitting problem and greatly reduce the validation error. Gao et al. [20] introduced a novel double-branched and area-constraint FCN structure to learn the shallow coarse and deep representations from the spectral domain OCT images for the segmentation of the SRF and PED regions. Xing et al. [36] proposed a new model based on FCN framework that included the attention gate and spatial pyramid pooling modules to better extract the multiscale objects in OCT images. A curvature regularization term was used to set the loss function to incorporate shape prior information and tune the network parameters, especially when the lesion is vague. The modified FCN structure combined the softmax as the loss function during the model training process.

### 4.2. Deep Learning Paradigms with U-Net Backbone

The merits of the U-Net have been verified and demonstrated in plenty of publications [21,37,38] in biomarker segmentation research fields. This subsection reviews the modified networks based on the U-Net backbone for the applications of retinal fluid segmentation.

#### 4.2.1. Fine-Tuning on the U-Net Backbone

Inspired by the U-Net and DeconvNet, Roy et al. [37] proposed a novel network named ReLayNet for OCT retinal layer and fluid segmentations. The ReLayNet worked by encoding the input image data with a contracting path of convolutional blocks to learn the hierarchical contextual features, and by decoding with an expansive path of convolutional blocks to generate the semantic segmentation results. The ReLayNet optimized a joint loss function that combined weighted logistic regression and Dice overlap loss for training the ReLayNet. Roy et al. [37] reported the excellent retinal layer segmentation results in terms of Dice overlap score, estimated contour error for each layer, and the error in estimated thickness map provided by the ReLayNet.

Kang et al. [38] used two fine-tuned U-Net networks for the IRF, SRF, and PED retinal fluid segmentation. The first network added a fully connected layer between the encoding and decoding paths of the U-Net in order to segment the original image and output the corresponding categories of the segmented area. The second U-Net involved both the OCT image and the corresponding segmented image generated from the former network as input data (two channels) to carry out further detailed segmentations.

The fine-tuned U-Net used in the work of Lee et al. [21] contained 18 convolutional layers and sigmoid activation functions in the last layer to output the binary segmentation probability. A vertical window size of 432 × 32 pixels was selected to perform sliding on the image. The U-Net computed the window patch inputs to generate a probability distribution map which can be finally visualized to illustrate the IRF segmentation regions.

#### 4.2.2. Loss Function Innovations

A good loss function not only may improve the accuracy of segmentation, but it could also help speed up the training process,. The research on the loss function design has been attracting attention from the community. Tennakoon et al. [39] introduced the adversarial loss as the U-Net loss function, which can encode the higher-order relationships between image regions. Furthermore, they considered the batch normalization to each of their convolution blocks to ameliorate training efficiency, and also employed the dropout for each jump connection to prevent overfitting. Their proposed method achieved the fourth-best score listed in the RETOUCH challenge result panel.

Liu et al. [40] proposed a semi-supervised method with an adversarial learning strategy. The segmentation network and a discriminator network were constructed with fully convolutional architectures. Their major contribution was to optimize the joint loss function as the objective function, which integrated the multi-class cross-entropy loss, Dice overlap loss, adversarial loss, and semi-supervised loss [40]. Such an adversarial learning strategy just required much smaller numbers of retinal fluid labels for network training.

Wei and Peng [41] utilized the priority of the mutex relationship between different layers to adjust the Dice loss function to be mutex Dice loss, to better segment retinal layers and fluids in OCT images. Moreover, they proposed a depth max pooling operation, which selected the maximal feature map from the up-pooling layer and the corresponding convolutional layer in the encoder block. Such an operation made the channel and parameters remain unchanged, which can avoid the doubling of the channel and training parameters caused by skip connections.

#### 4.2.3. U-Net with New Modules

Recently, new modules were employed in the U-Net construction, with the purpose of better coping with different sizes of retinal fluid and utilizing the spatial information of OCT images. Chen et al. [42] proposed a method that integrates the squeeze-and-excitation block (SE-block) [51] into the U-Net to segment the retinal fluid regions. The regions of interest (layers between internal limiting membrane (ILM) and BM) processed by the graph-based segmentation algorithm [52] were used as the U-Net inputs.

In the work of Hassan et al. [8], the SEADNet consisted of three modules, i.e., a feature encoder, two multiscale feature extractors, and a feature decoder, for segmentations of IRF, SRF, and PED. The basic structures of the feature encoder module and feature decoder module were the same as those in the classical U-Net. However, the batch normalization [53] (BN) was added in each convolution block to speed up training. The multiscale feature extractor module included a dense atrous convolution (DAC) block [54] and an ASPP block. The DAC block can ensure that the network effectively extracts the retinal fluid features of different sizes, and the ASPP block makes the network more robust in response to the contour, delineation, and geometry changes.

Similarly, Ma et al. [43] embedded the ASPP module into the FCN network, and combined U-Net to design a dual-branch neural network for retinal layer and retinal fluid segmentation. The encoder path of U-Net performed the feature map extraction of the input image. Meanwhile, the decoder path of U-Net and FCN restored the resolution of the feature map, respectively. The U-Net network was effective on coarse segmentation, but it had limited performance on boundary segmentation, whereas the FCN can reliably extract boundary pixels. Therefore, a combination of the U-Net and FCN could be a possible solution toward a higher segmentation accuracy.

Ye et al. [9] proposed the context pyramid guide (CPG) module and inserted it between the encoder and decoder paths, so that the network can dynamically fuse multi-scale information from high-level features. Their module design was inspired by the spatial pyramid structure [12], which can be used to collect the multiscale information. In order to effectively reduce the redundant feature maps, they proposed the context shrinkage encode module based on the soft thresholding function [55], and embedded it in each convolution block of the encoder path as the last layer in the deep learning network.

To better deal with the diversity of SRF, Yang et al. [23] replaced the conventional pooling layer in the U-Net encoder with six-string convolutions based on residual architecture. They proposed a multiscale pyramid pooling module with reference to the pyramid pooling module of DeeplabV3, and connected it behind the six-string convolutions.

Considering the inconsistent sizes of PED in different OCT images, Bao et al. [24] abandoned the skip connections of the U-Net and employed a channel multiscale module, to obtain the multiscale information with the attention mechanism. Moreover, a spatial multiscale module designed on the basis of dilated convolution was inserted into the decoder path, so that the deep learning network can compute more multiscale receptive fields with spatial information.

#### 4.2.4. 3D U-Net Model

In the previous work of Lin et al. [44], a BN layer was employed for each convolution block (Conv+BN+ReLu) with the focal loss function as well. In 2019, Li et al. [56] utilized the 3D U-Net network architecture to perform the IRF and SRF segmentations in OCT images. If a single OCT image cannot be desirably segmented, the 3D network architecture can gather the spatial feature information of the adjacent OCT images to improve the segmentation. In addition, they may overcome the overfitting obstacles and a few pixel segmentation difficulties.

### 4.3. Hybrid Computational Segmentation Methods

In addition to the deep learning methods mentioned above, the hybrid approaches are also widely used to combine several effective algorithms to segment and identify the retinal fluids in OCT images.

#### 4.3.1. Shortest-Path Methods

Rashno et al. [45] reported a fully automated method to segment and detect three types of retinal fluid. Their method considered the shortest-path graph technique to segment the ILM and RPE, and then took them as input data to train the CNN for binary classifications of the pixels between two regions. Then, the PED was segmented by combining the results of layer segmentation and flattening. Similar to the previous related studies, Liu et al. [46] added the BM3D method to denoise the image, and computed the shortest paths for the retinal fluid segmentation in the spectral domain OCT images. A type of rectangular box was also added to frame the content of the original image, in order to train the CNN for detection of retinal fluid locations.

#### 4.3.2. Graph-Based Methods

The FCNs were frequently used as the backbone structures for hybrid fluid region segmentation applications. Rao et al. [22] studied the negative impact of speckle-noise generated in OCT scanning on IRF segmentation. They used the IOWA reference algorithm to convert the area outside the ILM and RPE layers into the zero-intensity area, and then input the processed background neutralized image into the U-Net for segmentation. The IOWA reference algorithm can perform the graph-based simultaneous surface segmentations with varying constraints and regional information. Lu et al. [47] implemented the layer segmentation by using a graph-cut algorithm. In their study, the FCN was trained to segment all pixels of each B-scan image into the non-fluid areas and the regions of IRF, SRF, and PED, respectively. Then, features were extracted from the potential fluid regions, and a random forest classifier was used to reject the false fluid regions.

#### 4.3.3. Unsupervised Learning Methods

In 2017, Montuoro et al. [48] developed a novel automated 3D layer that incorporated the unsupervised representation, auto-context loop, and graph-theoretic segmentation to establish a retinal fluid segmentation system. Their method first extracted the image-based features from the original OCT image data, and combined the manual labels to train a basic voxel classifier. Then, the probability map was generated to perform the surface segmentation and extract the context-based features to train another classifier. The procedures of context-based feature extraction and additional classifier training were iteratively repeated multiple times in an automatic context loop.

Gopinath and Sivaswamy [49] trained a CNN with generalized motion patterns for retinal fluid segmentations in spectral domain OCT images. Their technique first selected the enhancement of the cyst regions prior to the network training. Then, a mapping function that combined the multiple motion results was optimized by the CNN to generate a probability map of the cyst’s position in a given OCT image. Finally, the cyst segmentation results were produced by clustering of the identified cyst locations.

He et al. [50] exploited the similarity between the retinal effusion fluid regions and background regions, and the dissimilarity of the retinal layers and background regions, to create an intra-and inter-slice contrastive learning network (ISCLNet) to accomplish the point-supervised retinal fluid segmentations in OCT images. It is a weakly-supervised learning method that can work with any CNN-based backbone, and may perform a rapid training of the ISCLNet model for retinal fluid segmentation.

## 5. Discussions

A wide variety of datasets lead to different evaluation criteria for retinal fluid segmentation. In order to better compare and discuss different segmentation methods reported in recent publications, we summarized the methods and their results on the same public datasets (i.e., RETOUCH, Duke, and OPTIMA) or multiple private datasets in Table 3 and Table 4. The segmentation performance of deep learning architectures on the public datasets are comparable. The retinal fluid segmentation results reported by researchers based on their private datasets may also be valuable for segmentation evaluations. According to Hassan et al. [8], the SEADNet that integrated the DAC and ASPP modules can achieve the best overall segmentation results on the the RETOUCH dataset, with the averaged Dice results of 90.9%, 91.3%, and 91.8% for the segmentations of IRF, SRF, and PED regions, respectively. The dual U-Net proposed by Kang et al. [38] also achieved the comparable OCT segmentation results of IRF, SRF, and PED, with the Dice values of 86.00%, 92.67%, and 91.63%, respectively. It can be observed that the best Dice result of 95.78% for the single PED segmentation on the RETOUCH dataset was obtained by the FCN-based RetFluidNet, reported in the work of Sappa et al. [32]. In addition, the depthwise separable CNN proposed by Girish et al. [33] provided the best IRF segmentation accuracy in terms of Dice value of 74% on the OPTIMA dataset, which is similar to the IRF segmentation Dice result of 76% on the Duke dataset, as reported by Montuoro et al. [48].

Regarding the segmentation results obtained with the deep learning architectures based on the CNN-based or FCN-based backbones, the FCN method of Schlegl et al. [19] can accurately distinguish fluid localization and extent, with the Dice values of 77% and 79% for IRF and SRF segmentations, respectively. Venhuizen et al. [27] combined two FCN models to automatically segment the intraretinal cystoid fluid and diffuse non-cystic IRF regions. The first FCN network can be used as the additional input to the second FCN, which can learn a wide range of different complex features and has the capability of capturing the large variability in AMD appearance and vendor-dependent spectral domain OCT characteristics. They reported a Dice value of 75.4% for IRF segmentation [27]. Liu and Wang [34] proposed an uncertainty-aware semi-supervised framework that consisted of a teacher network and a student network, with the same structure. Three decoders of each network can simultaneously output the probability map, contour map, and distance map. They reported that the fusion of these three maps may achieve the averaged Dice values of 73.6%, 75.57%, and 73.93% for IRF, SRF, and PED segmentations on the RETOUCH dataset, respectively.

Sappa et al. [32] and Hu et al. [26] both integrated the ASPP module in the FCN. Sappa et al. [32] reported that the FCN with ASPP module may provide the Dice results of 80.05%, 92.74%, and 95.53% for the IRF, PED, and SRF segmentations on the RETOUCH dataset, respectively. Hu et al. [26] reported that their deep learning network may achieve the Dice values of 87.59% and 79.71 % for the SRF and PED segmentations in spectral domain OCT images, respectively. In addition, Chen et al. [28] used the modified faster R-CNN architecture for automatic retinal fluid segmentation, with the Dice value of 70.44% when detecting the IRF regions.

The combinations of additional computational modules and CNN-based or FCN-based backbones are also popular, because these computational methods could bring about reducing training expense or increasing feature diversity for segmentation accuracy improvements. Pawan et al. [25] utilized the dilation, residual connections, inception blocks, and capsule pooling in their model, which could decrease the number of trainable parameters by 37.85%, with a competitive Dice result of 94.04% for SRF segmentation. The depthwise separable convolutional filter method proposed by Girish et al. [33] facilitates model generalization and prevents model overfitting, and leads to a Dice value of 74% for IRF segmentation.

The optimizations of deep learning networks could effectively improve the instance segmentation performance. The U-Net with 18 fine-tuned convolutional layers proposed by Lee et al. [21] can successfully segment the IRF regions up to the Dice of 72.90%, when handling the OCT images with different characteristics. Tennakoon et al. [39] used an adversarial loss function to avoid the image post-processing operations, and achieved the Dice values of 69.00%, 67.00%, and 85.00% for the IRF, SRF, and PED segmentations, respectively. Wei et al. [41] proposed a deep max pooling scheme to reduce the network parameters and speed up the model training procedure. They reported that the FCN with the deep max pooling scheme may reach a Dice value of 81.00% in retinal fluid segmentations on the Duke dataset. In the past three years, several new modules were utilized into the U-Net backbones to obtain the multiscale spatial feature information of retinal fluids. The six striding convolutions designed by Yang et al. [23] provide more possibilities for segmenting retinal fluid of different sizes, and their network could provide a Dice value of 96.20% for the SRF segmentations. In order to extract multiscale features from the OCT images, the CPG module was introduced by Ye et al. [9], and the channel multiscale module was used by Bao et al. [24] to replace the skip-connection layer of the U-Net. Ye et al. [9] reported that the Dice values of 73.17%, 79.10%, and 71.06% could be obtained in the IRF, SRF, and PED segmentations on the RETOUCH dataset, respectively. Bao et al. [24] reported a PED segmentation Dice result of 79.17% on their own OCT dataset.

Hybrid methods usually emphasize the preprocessing steps, and tend to combine different networks or computational algorithms toward better segmentation results. Rashno et al. [45] and Liu et al. [46] introduced the shortest path as the input data of the CNN-based backbones during the preprocessing process, and their hybrid methods can achieve the Dice coefficient results over 80% both in the semantic and instance segmentations of retinal fluid regions. Rao et al. [22] reported that the preprocessing step could greatly reduce the noise interferences during the FCN model training, and the conventional feature extraction techniques may assist in the SRF segmentations, with a Dice coefficient value over 90%. Lu et al. [47] focused on the prior, domain-specific knowledge in the graph-cut algorithm, which could help the Dice performance of the deep learning architecture exceed 70% for the IRF, SRF, and PED instance segmentations, on the RETOUCH dataset.

The unsupervised learning and weakly supervised learning algorithms could also provide excellent retinal fluid segmentation accuracy results. In the research of Montuoro et al. [48], the unsupervised learning method was combined with the auto-context and graph theory for the retinal fluid segmentation applications. Such a method may achieve the Dice coefficients of 76% for both of the IRF and SRF segmentations on the Duke OCT dataset. According to Gopinath and Sivaswamy [49], although the convolutional network trained by the unsupervised learning algorithms was unable to detect very small cystic areas in the OCT images, the unsupervised learning approach still could implement the IRF segmentation with a Dice value over 71% on the OPTIMA dataset. He et al. [50] applied a contrastive weakly learning network to the field of retinal effusion segmentation, and accomplished the Dice values of 73.16% and 69.42% for SRF and PED segmentations, respectively, which were superior to the results of the other well-known point-supervised methods.

Regarding the semantic segmentation performances, Ma et al. [43] used the FCN with the ASPP module as an extension path to ameliorate the capability of the U-Net backbone for boundary segmentations of both retinal layer and retinal fluid segmentations, and reported the Dice result of 51.32% for semantic segmentations. In the work of Sanchez et al. [29], the SEUNet model ensembled with the modified deep retinal understanding framework and a Pix2Pix module was able to obtain the Dice segmentation result of 62.53% when detecting small lesions. The ReLayNet proposed by Roy et al. [37] considered the weighted logistic regression in the loss function, and segmented the retinal fluid regions with a Dice result of 77%. Based on the idea of generative adversarial networks [57], Liu et al. [40] designed the U-Net backbone with a discriminative network for segmentation, which could achieve 80.00% Dice coefficient on retinal fluid segmentation, by training with several labeled data and unlabeled data. Chen et al. [42] used the SE-block module in the U-Net and made the semantic fluid segmentation with an excellent Dice of 94.21%. Wang et al. [30] integrated the ASPP module in their deep learning architecture to detect macular edema, and used the CRF to refine the macular edema boundary for learning a wide variety of different complex features of OCT images. Their proposed method achieved a Dice value of 95.43% for the retinal fluid segmentation.

## 6. Conclusions

This article provides a comprehensive survey on the recent significant academia’s contributions to the segmentation of retinal fluid in OCT images based on deep learning architectures. The majority of deep learning architectures were established based on the CNN, FCN, U-Net, and hybrid methods. The OCT images in the same dataset can come from different vendors, which would affect the retinal fluid segmentation results. Therefore, future works should focus on domain generalizations to eliminate the dependence of segmentation on the dataset.

The advanced deep learning methods have several advantages for the accurate and effective detection of retinal fluid lesions, which can better support the diagnosis decision making of ophthalmic diseases in more clinical applications. Furthermore, the automatic retinal fluid segmentation may prevent the subjective influence of manual detection and reduce the workload of medical experts [58]. With the developments of advanced deep learning technology in the future, the retinal fluid segmentation methods based on the deep learning architectures will progressively replace the manual segmentation protocol in clinical practice.

## Figures and Tables

**Table 1:** Public benchmark optical coherence tomography (OCT) datasets for retinal fluid segmentation. AMD: Age-related macular degeneration, CNV: choroidal neovascularization, DME: diabetic macular edema, IRF: intraretinal fluid, MS: multiple sclerosis, PED: pigment epithelial detachment, RVO: retinal vein occlusion, SRF: subretinal fluid. Dataset web pages last accessed on 9 February 2022.

Dataset	Data Size	Manual Labeling	Disease	Web Page
RETOUCH	70 volumes	IRF, SRF, PED	AMD, RVO	https://retouch.grand-challenge.org
UMN	600 B-scan images	IRF, SRF, PED	AMD	http://people.ece.umn.edu/users/parhi/data-and-code/
OPTIMA	30 volumes	IRF	AMD, RVO, DME	https://optima.meduniwien.ac.at/optima-segmentation-challenge-1/
Duke	110 B-scan images	Fluid regions	DME	https://people.duke.edu/~sf59/Chiu_BOE_2014_dataset.htm
HCMS	49 B-scan images	Fluid regions	MS	http://iacl.jhu.edu/Resources
Kermany	108,312 B-scan images	Fluid regions	CNV, DME, drusen	https://data.mendeley.com/datasets/rscbjbr9sj/2

**Table 2:** Deep learning architectures and paradigms for retinal fluid segmentation. ASPP: atrous spatial pyramid pooling, CNN: convolutional neural network, DAC: dense atrous convolution, FCN: fully convolutional network.

Deep Learning	Methodology	Research Works	Paradigms
		Schlegl et al. [19]	8-layer FCN
		Chen et al. [28]	Faster R-CNN
	Modified CNN	Sanchez et al. [29]	SEUNet model
		Wang et al. [30]	Deeplab model
		Sappa et al. [32]	CNN (skip-connect operations + atrous spatial pyramid pooling)
FCN backbones		Girish et al. [33]	Enhanced semantics preprocessing + CNN with a depthwise separable convolution filter
		Liu and Wang [34]	Two (Student + Teacher) FCNs
		Pawan et al. [25]	DRIP-Caps (SegCaps with dilation, residual connections, inception blocks, and capsule pooling)
	Modified FCN	Hu et al. [26]	ResNet added with ASPP and modified stochastic ASPP
		Gao et al. [20]	FCN with modified loss funciton
		Xing et al. [36]	FCN architecture with attention gate and spatial pyramid pooling module
		Roy et al. [37]	ReLayNet optimized with the weighted logistic regression and Dice loss
	Fine-tuning	Kang et al. [38]	A cascade of two fine-tuned U-Nets (the second U-Net with 2-channel inputs)
		Lee et al. [21]	U-Net with 18 convolutional layers and sigmoid activation function for probability distribution mapping
		Tennakoon et al. [39]	Adversarial loss
	Modified loss function	Liu et al. [40]	Semi-supervised loss
U-Net backbones		Wei and Peng [41]	Mutex Dice loss
		Chen et al. [42]	Squeeze-and-Excitation block (SE-block)
		Hassan et al. [8]	Multiscale feature extractor module (DAC + ASPP)
	Functional modules	Ma et al. [43]	U-Net + FCN with ASPP module
		Ye et al. [9]	Context pyramid guide + Context shrink-age encode
		Yang et al. [23]	Six-string convolutions + Multiscale pyramid pooling module
		Bao et al. [24]	Channel multiscale module + Spatial multiscale module
	3D U-Net	Lin et al. [44]	Batch normalization + Focal loss
	Shortest path methods	Rashno et al. [45]	Graph shortest path + CNN
		Liu et al. [46]	BM3D method + Graph shortest path + CNN
	Graph-based methods	Rao et al. [22]	IOWA reference algorithm + U-Net
Hybrid methods		Lu et al. [47]	Graph-cut algorithm + FCN + Random forest
		Montuoro et al. [48]	Unsupervised representation + Auto-context loop + Graph-theoretic segmentation
	Unsupervised learning algorithms	Gopinath and Sivaswamy [49]	CNN trained with generalized motion patterns + Clustering
		He et al. [50]	Intra- and inter-slice contrastive learning network (ISCLNet) + CNN

**Table 3:** Summary of important results of the IRF, SRF, and PED segmentations. The Dice results of IRF, SRF, and PED were the mean value of OCT image segmentation provided by different OCT vendors. “N/A” indicates that this type of fluid is not segmented in the paper or is not segmented by the method of deep learning.

Research Work	Dataset	Backbone	Loss Function	IRF	SRF	PED
				Dice (%)	Dice (%)	Dice (%)
Hassan et al. [8]	RETOUCH	U-Net	Dice loss	90.90	91.30	91.80
Ye et al. [9]	RETOUCH	U-Net	Combination of cross-entropy and Dice loss	73.17	79.70	71.06
Schlegl et al. [19]	Private	FCN	Softmax loss	77.00	79.00	N/A
Gao et al. [20]	Private	FCN	Combination of area and softmax loss	N/A	95.30	91.90
Lee et al. [21]	Private	U-Net	ReLu, sigmoid loss	72.90	N/A	N/A
Rao et al. [22]	Private	FCN	ReLu, sigmoid loss	N/A	91.00	N/A
Yang et al. [23]	Private	U-Net	Combination of binary cross-entropy, Dice, and diff loss	N/A	96.20	N/A
Bao et al. [24]	Private	U-Net	Combination of binary cross-entropy and Dice loss	N/A	N/A	79.17
Pawan et al. [25]	Private	SegCaps	Margin loss	N/A	94.04	N/A
Hu et al. [26]	Private	ResNet50	Cross-entropy loss	N/A	87.59	73.71
Venhuizen et al. [27]	Private	FCN	Weight map loss	75.40	N/A	N/A
Chen et al. [28]	RETOUCH	Faster R-CNN	Softmax and smooth L1 loss	70.44	58.83	70.31
Sappa et al. [32]	RETOUCH	FCN	Combination of softmax and cross entropy loss	78.95	90.90	95.78
Girish et al. [33]	OPTIMA	DSCN	Weight map loss	74.00	N/A	N/A
Liu et al. [34]	RETOUCH	FCN	Combination of Dice loss, cross-entropy, regression and consistency loss	73.60	75.57	73.93
Xing et al. [36]	RETOUCH	FCN	Shape prior loss function	79.00	74.00	77.00
Kang et al. [38]	RETOUCH	U-Net	Maxout loss	86.00	92.67	91.33
Tennakoon et al. [39]	RETOUCH	U-Net	Combination of cross-entropy, Dice loss, and adversarial loss	69.00	67.00	85.00
Rashno et al. [45]	RETOUCH	CNN	ReLu, tanh loss	85.45	81.30	84.08
Lu et al. [47]	RETOUCH	FCN	Combination of softmax and cross entropy loss	71.90	75.53	72.06
Montuoro et al. [48]	Duke	Unsupervised 3D-layer	Probability map	76.00	76.00	N/A
Gopinath et al. [49]	OPTIMA	CNN	Weight binary cross entropy loss	71.00	N/A	N/A
He et al. [50]	RETOUCH	ISCLNet	Inter-slice loss, cross-entropy loss, and intra-slice loss	N/A	73.16	69.42

**Table 4:** Summary of important results of retinal fluid segmentation, without further segmentation of IRF, SRF, or PED regions. The Dice results are sorted in ascending order. SEUNet: U-Net with squeeze-and-excitation blocks, DRIU: deep retinal understanding.

Research Work	Dataset	Backbone	Loss Function	Fluid in Dice (%)
Ma et al. [43]	Private	U-Net+FCN	Combination of weighted Dice loss and the weighted logistic loss	51.32
Sanchez et al. [29]	Duke	Ensemble network (SEUNet, DRIU)	Cross-entropy, Dice, Jaccard loss	62.45
Roy et al. [37]	Duke	U-Net	Combination of weighted logistic regression and Dice loss	77.00
Liu et al. [40]	Duke	U-Net	Combination of cross entropy, Dice, adversarial, and semi-supervised loss	80.00
Liu et al. [46]	Private	CNN	Relu and softmax loss	81.10
Hao et al. [41]	Duke	U-Net	Combination of weighted cross-entropy, Dice and mutex Dice loss	81.00
Chen et al. [42]	Public	U-Net	Dice loss	94.21
Wang et al. [30]	Private	DeepLab	Cross-entropy loss	95.43
Li et al. [56]	Private	3D U-Net	Combination of weighted loss and focal loss	95.50

## Abbreviations

The following abbreviations are used in this manuscript:

AMD	Age-related Macular Degeneration
ASPP	Atrous Spatial Pyramid Pooling
BM	Bruch’s Membrane
BN	Batch Normalization
CNN	Convolutional Neural Network
CNV	Choroidal NeoVascularization
CPG	Context Pyramid Guide
CRF	Conditional Random Field
DAC	Dense Atrous Convolution
DME	Diabetic Macular Edema
FCN	Fully Convolutional Network
GPU	Graphics Processing Unit
GRU	Gated Recurrent Unit
ILM	Internal Limiting Membrane
IRF	Intraretinal Fluid
OCT	Optical Coherence Tomography
PED	Pigment Epithelial Detachment
RPE	Retinal Pigment Epithelium
RVO	Retinal Vein Occlusion
SE-block	Squeeze-and-Excitation block
SRF	Subretinal Fluid
